# Risk Entanglement in the Finance-State Nexus: The Case of Systemic and Political Risk

**DOI:** 10.3389/fsoc.2022.877217

**Published:** 2022-04-25

**Authors:** Markus Lange, Christian von Scheve

**Affiliations:** ^1^Institute of Sociology, Martin Luther University of Halle-Wittenberg, Halle, Germany; ^2^Institute of Sociology, Free University of Berlin, Berlin, Germany; ^3^German Institute for Economic Research (DIW) Berlin, Berlin, Germany

**Keywords:** finance, state, risk, relationality, systemic risk, political risk

## Abstract

Crises such as European debt crisis, Brexit, and COVID-19 have challenged established relations between finance and the state in attempts at mitigating a broad range of crises-related risks. We ask whether and how these altered relations in themselves constitute novel uncertainties and risks between the two fields. To better understand these dynamics, we introduce the concept of “risk entanglement” to complement financialization as a key concept presently capturing these relations. Based on qualitative research in the German finance-state nexus, we show how financial and state actors mutually construe each other as risks that need to be managed and mitigated to safeguard their particular, field-specific logics and ends. We focus on systemic risk and political risk as two cases of risk entanglement: whereas systemic risk reflects the threat of a potential financial meltdown to the state, political risk reflects how the state endangers established risk practices in finance.

## Introduction

The era since 2007 is best characterized as an era of crises, involving financial and state actors. This includes not just the global financial crisis of 2007–2009 and the European debt crisis starting in 2010, but also Brexit and the COVID-19 pandemic. Not only did these encompass finance and state actors in their own right, but the very financial, fiscal, regulatory, and political relations between finance and statehood served as both catalysts inciting and assets curtailing these crises. Regarding the pandemic, for example, the Next Generation EU recovery plan provides for joint debt borrowing and joint liability of its members, demonstrating an initial deviation from the paths taken during the sovereign debt crisis. This particular constellation led political commentators and researchers alike to assert that the relations between financial and state actors are crisis-driven, having become dysfunctional by any measure (Tooze, 2018).

Economic sociology and political economy investigate these crisis-driven relations predominantly with regard to processes of financialization (van der Zwan, 2014) affecting state practices (Braun et al., 2018), public debt management (Woodruff, 2016; Fastenrath et al., 2017), central banks as finance-state hybrids (Kalaitzake, 2019; Braun, 2020; Walter and Wansleben, 2020), the supervision and regulation of finance (Epstein and Rhodes, 2016b; Hardie and Macartney, 2016), and statist issued financial assets (Gabor, 2016; Gabor and Ban, 2016), to name some examples. In a nutshell, this research shows that the logics and interests of financial markets continue to proliferate and influence statehood, even in an era of crisis and enduring calls for a “strong state.”

Receiving considerably less attention is the fact that the implications of crises for finance-state relations are not unidirectional and that statehood is, in principle as well as in times of crisis, a significant factor for change and transformation in finance. Although there is a vast body of literature on the role of states and governments for financial markets, considerably less literature specifically investigates the changing dynamics of the mutuality of state-finance relations in times of crisis. This contribution aims to fill this gap by arguing that a more in-depth understanding of the dynamics of the finance-state nexus (FSN), especially—but not exclusively—during crises, can be achieved by focusing (a) on the distinct, field-specific practices of state and financial actors; (b) on their common and intertwined practices; and (c) on how both are challenged, transformed, and reconfigured through crisis-induced social, political, and economic change.

Arguably, this is a vast undertaking, which is why we focus, as an initial step, on whether and how the manifold crisis-afflicted relations between finance and statehood have brought about novel (and mutual) uncertainties, dangers, and risks. Our emphasis on risk and risk-related concepts is motivated by the fact that responses to crises typically involve the assessment and management of risk, with both finance and statehood well-known for dealing with dangers in a risk-oriented fashion (Luhmann, 1993). Typically, risk is understood as a specific quality of situations involving decisions with uncertain, potentially harmful, outcomes and as a phenomenon experienced by an individual decision maker or ascribed to a situation by an observer. To live up to our proposed emphasis on crisis-driven relational dynamics between statehood and finance, we take a slightly different approach, advocating for a relational understanding of risk (Boholm and Corvellec, 2011). This understanding capitalizes on how social actors (or fields, institutions, etc.) can become “risk entangled” as a consequence of mutually assessing one another as “risk objects.”

From this vantage point, we ask to what extent financial and state actors mutually construe each other as risks that need to be managed and mitigated in order to safeguard their own, field-specific logics and ends. How, in times of crisis, do state and government become a risk for finance, and, conversely, how is the financial world addressed as a risk in politics and governments? To address these questions, the present contribution presents results of research on the finance-state nexus in Germany between 2017 and 2020. To investigate risk relations between finance and state actors, we carried out 68 in-depth interviews with German banks, insurance companies, federal parliaments, state (Länder) parliaments, treasury departments, as well as financial supervisory and regulation authorities. This data is complemented by 1 year of ethnographic observations of public and closed meetings of the Finance Committee of the German Bundestag.

In the following, we first review research in economic sociology and political economy that focuses on interdependencies between finance and statehood. Following developments in the sociology of risk, in relational sociology and field theory, we then illustrate and further develop the concept of relational risk. Drawing on this conceptual primer, the third section then outlines our research design and the fourth section presents the results of our empirical analysis along the lines of systemic and political risk. We close with concluding remarks in the fifth section.

## Toward a Relational Risk Perspective on Finance and Statehood

In the 2010s, scholars in economic sociology and political economy put increasing emphasis on the crises-related transformations of interdependencies between finance and statehood. Reviewing this research in the first part of this section shows that the FSN is typically considered a “financialized social sphere.” State actors adapt financial logics to perpetuate their own interests and legitimations, which can subsequently stabilize and reproduce financial practices. Although these approaches cover important relations between finance and statehood, we argue that exclusively focusing on financialization as a longer-term process misses important dynamics between the two fields. In short, financialization research focuses on how financial logics affect state practice (which may rebound to finance), while other, non-financialized facets of state practices affecting finance remain neglected. Thus, in the second part, we introduce the concept of “risk entanglement” to account for the co-construction of financial and state practices.

### The Finance-State Nexus in Economic Sociology and Political Economy

In the European Union, two fundamental shifts have altered the interplay between financial and state actors since 2010: First, and primarily as a consequence of the global financial crisis accompanied by the political initiative for a capital markets union (Rynck, 2016; Epstein and Rhodes, 2018), the European System of Financial Supervision (ESFS) was implemented as a new regulatory and supervision regime. Second, and contrary to these practices to restrict and oversee financial activity, the anticipation of the European sovereign debt crisis made it necessary to “calm” financial actors and maintain market-based finance activity (Hardie, 2013; Arjalies et al., 2017). This led to an era of expansionary monetary policy characterized by “quantitative easing” through bond purchase programs and low interest rates (Ronkainen and Sorsa, 2018).

Reflections of these transformations in economic sociology and political economy argue that financialization, understood as the expansion of market-based financial logics to other economic and societal areas (van der Zwan, 2014), is less contained through these interventions but is, in part, an essential component of them. Basically, scholars elaborate on the historical roots of financialization (Krippner, 2011; Davis and Walsh, 2016), on economists as a prestigious expert group (Hirschman and Berman, 2014), or on the implementation of economic models and metrics for financial and likewise non-financial policies (Heimberger et al., 2020; Angeletti, 2021).

Therefore, scholars who focus the political project of a capital market union in the European Union speak of a “governing through financial markets” (Braun et al., 2018), which, for them, means “to engineer and re-purpose financial instruments and markets as instruments of statecraft, with the goal of achieving economic policy goals at minimum fiscal cost” (Braun et al., 2018, p. 104). Woodruff (2016) describes this even more drastically, speaking of a “governing by panic”: State actors force market-based policies especially in crisis periods, which is examined specifically with regard for Germany's role during the sovereign debt crisis in enforcing austerity politics (but see Epstein and Rhodes, 2016a for a contrarian view on Germany's role). Financialized statehood is then confronted with the implementation of financial logics, on the one hand, and the potential threatening of its sovereignty on the other (Karwowski, 2019). The governance of financial fields is, as Konings (2015) puts it, “forever plagued by its embeddedness: the impossibility of cleanly extricating itself from the processes that it seeks to regulate” (Konings, 2015, p. 273). In the following, we detail this with respect to the regulation and supervision of finance, the management of state budgets and public debt through finance, and the “hybrid” role of central banks.

The institutionalization of the ESFS aims at consolidating the diversity of national supervision authorities and regulation practices within a single European framework (Epstein and Rhodes, 2016a; Lombardi and Moschella, 2016). This has led to new challenges, for example when national actors advocate the protection of their “national banking champions,” thereby counteracting the new regulatory imperative of stronger regulation of too-big-to-fail organizations (Epstein and Rhodes, 2016b; Hardie and Macartney, 2016). In principle, like Thiemann and Lepoutre (2017) show, to better grasp financial compliance or avoidance, it is not sufficient to focus on market actors alone, but one also needs to look at their relationships with regulators.

Overlooking the management of state budgets and public debt also reveals marked-based and financialization processes. This concerns austerity politics, like the limitation of public debt through market discipline (Bryan and Rafferty, 2017; Hood and Himaz, 2017; Ban and Patenaude, 2019). However, practical public debt management also follows this logic (Hardie, 2012; Paudyn, 2014). According to Fastenrath et al. (2017), market-based instruments (e.g., government bonds) offered to financial actors through auctions represent such a financialized debt management. Consequently, these financing practices can restrict state sovereignty. Analyzing “repurchase agreements” (repos), Gabor and Ban (2016) show that the use of government bonds as collateral for such repos would result in a shift in state-finance relations if the reputation of states now depends on this collateral quality.

Finally, the current role of central banks, in which financial supervision and public debt management crystallize, is critical for financialization. For example, not only is the European Central Bank (ECB) now the supreme supervisory authority, it also contributes to the maintenance of finance activity through bond purchase programmes and low interest rates. Thus, existing research discusses to what extent central banks maintain their pre-crisis mantra of market neutrality and price stability as the yardstick of their ex-post crisis mode, but ultimately act increasingly politically (van't Klooster and Fontan, 2019). This is flanked by influential financial sector interests (Kalaitzake, 2019; Walter and Wansleben, 2020). As Braun (2020) points out, while focusing repos and the (re)introduction of “simple, transparent, and standardized” securitisation practices as pillars of the current ECB monetary policy, financial actors do not only exercise instrumental power through lobbying, but also infrastructural power. Through their presence in respective ECB departments, they influence the design of these vehicles. This produces “infrastructural entanglement” between central bankers and financial actors that is separated from the parliamentary structure of the European Union.

Taken together, research on financialization provides a detailed account of the interdependencies between the state and the financial sector, with a strong focus on how political governance is influenced, and sometimes undermined, by financial knowledge, calculations, interests, and power. Although this account tends to neglect the extent to which core state and political logics also affect financial realities, its relational perspective provides a fertile starting ground for further analyzing how state and financial actors mutually construe each other as risks, especially in times of crisis. Thus, in the following section, we further develop this approach by introducing the concept of “risk entanglement” (von Scheve and Lange, under review) as a tool to understand how, with regard to risk, financial practices affect statehood and vice versa.

### Risk Entanglement

Finance and statehood can be conceived of as prototypical risk spheres that are equally confronted with dangers that either emerge from their societal (or ecological) environments or as parts of their germane operations and logics (Luhmann, 1993).

First, to capture the multi-layered relations between finance and statehood, we follow social constructionist perspectives of risk (Douglas and Wildavsky, 1982), drawing on Boholm and Corvellec's relational risk theory (2011) as a particularly fruitful starting point for our analysis. They distinguish “risk objects,” which are perceived as hazardous, from “objects at risk,” which are imbued with value and endangered by risk objects. In this approach, relationality means to practically establish “semantic” relations between these two objects, for example through the use of narratives, probabilities, or models. This likewise marks the social dimension of a contingent risk concept, because the way in which an actor constructs such a relation is framed by her internalized knowledge, standards, or experiences; this will probably vary across actors.

Second, our understanding of relational risk also accounts for social risk processing as a form of relationality. This mainly refers to social interactions in which actors confront each other by way of their risk seeking or risk avoidance strategies; for example, when communicating, calculating, or negotiating risk. Perspectives from relational sociology are useful to conceptualize the mutuality, the “in-between,” or the co-construction of risk relationships, for example by focusing on “trans-actions” between actors (Emirbayer, 1997) or the interplay of social interactions, relations, and networks (Crossley, 2011).

Third, we assume that perceptions, assessments, and the management of risk consolidate into what can be dubbed “risk practices,” which vary between different social fields, becoming relevant in interactions within and across fields, whether complementary and conflicting. To substantiate this view, we draw on Fligstein' and McAdam's understanding of “strategic action fields” (2012). On the one hand, strategic action fields are understood as “mesolevel social order” (Fligstein and McAdam, 2012, p. 3) in which interacting parties share common, but likewise contested and not fully determined practices, rules, and logics to coordinate (risk related) behavior. On the other hand, the concept of strategic action fields promotes an understanding of the manifold relations between fields, for example state- and non-state fields. This allows for a perspective on risk-related encounters between actors in different fields, where each actor embodies field-specific as well as common or shared risk practices.

With respect to the FSN, this conception suggests at least two distinct ideal typical risk categories: First, risk objects can emerge either in the social or ecological environment of finance and statehood, such as a nuclear catastrophe, pandemics, or market failures in the real economy, or within the FSN, such as financial or debt crises. Financial and state actors in both cases are affected by a common risk object, but will react in distinct ways to retain their specific objects at risk. Hence, it is plausible that these specific ways of addressing a common risk object may simultaneously challenge or transform the financial, fiscal, or regulatory interdependencies of finance and statehood.

The second category encompasses constellations of risk entanglement in which financial and state actors mutually construct each other as risk objects, while each actor retains their distinct objects at risk. Typically, these constellations occur in view of prolonged and institutionalized interactions, mutual dependencies, and contingencies between actors and/or fields. Actors then account for each other not only based on their goals, interests, and preferences, but also by considering the mutual relations they maintain with each other.

Thus, this perspective is well-suited for understanding relations within the FSN as constellations of risk entanglement, in times of crisis but also with regard to enduring, well-established relations. Financial actors, for example, aim at implementing and distributing financial instruments that generate profit and liquidity. To safeguard these financial objects at risk, they also become active politically, for example when seeking to contain or participate in designing regulatory interventions into financial markets (e.g., Hirschman and Berman, 2014; Thiemann and Lepoutre, 2017; Braun, 2020). In contrast, state actors aim at passing collectively binding policies, for which they need to maintain positions in power that are the fundamental objects at risk to them. To maintain these positions, state actors frequently engage in financial markets to refinance household budgets, for example by issuing government bonds. In the following sections, we use this field-oriented concept of risk entanglement to better understand the mutual interdependencies between the state and the financial sector in times of crisis.

## Methods, Research Field, and Data

An empirical investigation of relational risk in the FSN generally needs to focus on mutual observations, attributions, expectations, and risk practices, which are constructed, reproduced, and changed in the course of interactions and through social relations. Therefore, we pursue a relational-comparative approach as propagated by Grounded Theory (Clarke, 2009; Corbin and Strauss, 2015). Consequently, we use a common methodological repertoire not only to investigate financial and state actors, but also to be able to reconstruct distinct risk practices and risk entanglements. We conducted a qualitative study of the German FSN between 2017 and 2020 using narrative interviews and ethnographic fieldwork. We conducted 68 narrative problem-centered interviews (Witzel and Reiter, 2012) of financial and state actors. This method allows for accessing respondents' professional biographies, especially their crises experiences and reflections as well as related risk management concepts. We were also able to uncover relational and risk-related dimensions, for example, financial actors articulating attributions and action tendencies that result from their perceptions of, and interactions with, state actors—and vice versa.

A total of 25 interviews were conducted in the German financial sector, for example with actors from risk management, bond trading, structuring/derivatives, quantitative engineering, and research. We conducted interviews with public banks, private banks, and insurance companies, roughly covering the structure of the German financial sector. A further 26 interviews were conducted of members of parliament at both the German federal and federal-state levels. Here, respondents are politicians dealing with financial issues, like the regulation of the financial sector or management of public budgets and debt. Respondents mainly were members of the Finance and the Budget Committee of the German Bundestag. As contrasting cases, we also interviewed politicians from non-financial areas, such as ecological or cultural politics.

To better grasp the intertwining of finance and statehood, we also conducted 17 interviews of state financial-executives, for example with regulators, supervisors, and analysts at the German financial supervisory authority (BaFin), the Deutsche Bundesbank, and with treasurers at the federal and federal state levels who are involved in the issuance of government bonds. We also carried out 1 year of ethnographic fieldwork in public and non-public meetings of the Finance Committee of the German Bundestag. This allowed us to focus on the situational-interactional dimensions of risk between finance and statehood. All analyses were conducted using MAXQDA software.

## The Case of Political and Systemic Risk

In this section, we elaborate how financial and state actors construe each other as risk objects potentially threatening their respective objects at risk. We suppose that crisis management in the FSN has led to the manifestation of two risk types, systemic risk and political risk. Systemic risk reflects the threat for parliamentary and regulatory state actors of a potential collapse of the financial sector. In principle, preventing such a collapse will secure state actors' positions in power, which are the main objects at risk in the political field. Political risk refers to how financial actors conceive state actors and politics as threats to their established risk routines. Here, statehood, as risk object, endangers the securing of ongoing payments, liquidity, and, thus, profit margins as the key objects at risk in finance.

### Systemic Risk

In principle, post-crisis management in the observed German state field (and its relations to the European level) can be captured by the notion of systemic risk. The political claim to prevent a new financial crisis is closely related to the controlled containment of systemic financial risk. Two dominant dimensions become apparent here that are in line with general and finance-specific understandings of systemic risk (Renn et al., 2019; Schweizer, 2019): First, the danger of an overall loss of solvency with respect to market positions and outstanding obligations that concerns a nationally, supra-nationally, or globally approached financial sector. Second, financial cascade effects known as “interconnectedness,” which occur when debtors are no longer able to serve their obligations, which can, in turn, trigger payment deficits or even defaults by other financial actors as well. Thus, it is the combination of both dimensions that represents the danger of a system-wide failing of a financial sector and its markets^1^.

#### Finance as Risk Object

Following this basic understanding, to label a risk as “systemic” already represents it as a basic risk object: whereas typical risk types in finance address particular assets, actors, or properties associated to them (e.g., credit risk of a company, interest rate risk of a bond), the concept of systemic risk addresses an entire financial field. This is particularly evident in the statist financial-executive where to actively “work” with systemic risk is part of a new post-crisis induced self-understanding. The new role of regulators and supervisors is to actively search and minimize risks in finance that have a spill-over potential and thus a systemic quality. The following quote from a supervisor illustrates this:

Yeah, I mean that's our job, to search for risks. So we get paid to care.

Generally, with respect to the construction of finance as a risk object, several calculative practices (Callon and Muniesa, 2005) for measuring systemic risk have emerged (Adrian and Brunnermeier, 2016; Giglio et al., 2016; Acharya et al., 2017; Engle, 2018). However, these calculative devices are framed by a more general shift from a micro-prudential to a macro-prudential model of financial oversight (Baker, 2013; Goodhart, 2015; Kranke and Yarrow, 2018). Following Baker (2013), at the core of the micro-prudential model lies the rejection of a “fallacy of composition,” namely that a financial system is considered as stable if their single actors are stable. Instead of this, the macro-prudential model focuses the financial system as more than the sum of its parts, thus switching from an individual to a system-wide perspective as the predominant supervisory and regulative impetus (focusing in particular on procyclicality, herding behavior, and externalities, Baker, 2013, p. 114–117). Therefore, one could ask if to actively adapt systemic risk follows the macro-prudential idea congruently. Hereto, in Kranke and Yarrow's (2018) conception systemic risk “constitutes less a property of certain institutions than a relational product; put differently, such risk travels through the ties, rather than residing in the nodes” (Kranke and Yarrow, 2018, p. 4). Their analysis of systemic risk handling in the Financial Stability Board (FSB) and the International Monetary Fund (IMF) demonstrates that it is precisely this focus on the relational “in between” that the two organizations fail to address in their implementation of systemic risk.

For both the calculative practices and the organizational processes of systemic risk assessment, we find an interplay between an urge to take a system-wide view and the confrontation with single and diverse entities. The following statement by an analyst from the field of macroprudential supervision represents some of the dimensions in this respect:

Well, in the past systemic risks used to be hidden.… Risks of systemic relevance. They were not labeled like this. Of course, we know that large institutions are more dangerous for the big picture than a small Volksbank. But that someone tries to describe precisely, what is systemic relevance? What are the risks involved? And discussing instruments of how to fence these risks, too. This is quite new. Hence, it is not that risks have changed, rather the view of these risks.

With the 2008 financial crisis as an inflection point, considering “risks with systemic relevance” became increasingly important. This goes beyond the purely proportional consideration of the size of financial organizations, rather incorporating an assessment of finance as a risk object. Therefore, the individual risks would not have changed, but the view of them. This is characterized by a dominance of the systemic as a central lens through which, in principle, other risks, like credit, liquidity, and interest rate risks, are also assessed. The change from the micro- to the macroprudential has apparently generated strong norms and habits in the field of financial supervision. This is clear from the respondent's further comments:

I always say the banking supervisory authority, the microprudential side, tries to prevent banks from committing suicide, that is, from riding themselves into the abyss. Then the macroprudential side tries to contain the weapons of mass destruction. Thus, preventing banks from taking others or the whole system down with them.… The micro-supervisor, he has no mandate to impose conditions on the bank…, because what the bank is doing right now, the business it has just invented and with which it earns money—forbidding this business will have consequences that make others suffer. Different mandates, micro macro. So I think you cannot get systemic risk completely out of the micro side.

This mission statement, interpreted as a hyperbole, differentiates the micro-prudential imperative, to prevent the single “suicide” of a financial actor, from the macro-prudential imperative, the vigilance of “mass destruction” in a whole financial field, generating an evaluation in favor of the latter. Thus, a conflict between these macro- and micro-views becomes apparent: Regulatory approaches take up the systemic idea, such as double proportionality, i.e., the intensive risk monitoring rather of big than of small institutions. Nevertheless, concrete supervision is often carried out as an individual audit. This is accompanied by a mandate problem if the micro-supervisor prohibits a business practice that could potentially endanger the system but would generate a margin at the respective institute. Beyond this, the dominance of systemic risk rooted in the macro-prudential approach is also represented by some of the surveyed regulators and delegates who consider the insolvency of individual institutions to be less relevant than the stability of the “system”.

#### Statist Objects at Risk

Based on these practices in assessing finance as a risk object, we now elaborate how this danger threatens statist objects at risk. A subsuming category of objects at risk in statehood is the legitimation of power and corresponding positions in parliamentary or bureaucratic hierarchies (Luhmann, 1993, p. 145–174). For the MPs interviewed, mitigating societal risks and securing common goods and welfare are typical concepts of their self-descriptions to encounter the loss of their electoral mandate. Related to this, the danger of a financial collapse brings out more specific objects at risk: One main concern of delegates is to avoid the repetition of the sovereign as liable guarantor in case of such a collapse. During a meeting of the finance committee at the German Bundestag, one delegate expressed this as follows:

The ultimate interest of the faction is: taxpayers should not be liable again.

Other objects at risk mentioned by politicians include, for example, the need for the functioning of finance to ensure liquidity and financial safeguards toward an unknown future for the real economy. Besides these societal consequences of a financial collapse, which call for political attention, a peculiar criterion of systemic risk is that statehood is directly involved in its potential emergence. For example, when issuing government bonds, state actors serve as financial market actors and become part of risk evaluations. Regulatory interventions to contain risks can also trigger new risks as second-order dangers (Holzer and Millo, 2006; Esposito, 2013), if financial actors develop circumvention strategies that unleash new regulatory uncertainties (e.g., migration to less regulated markets or over the counter transactions).

In particular, the meetings of the Finance Committee shed light on how systemic risk is approached against the background of its potential to threaten valuable objects at risk. In this respect, we now highlight, in more detail, that during the parliamentary process another danger can arise, namely the collective failure to correctly evaluate systemic risk. Although finance is identified as a risk object, parliament can fail to make decisions aimed at mitigating this risk. Besides complex negotiations along diverse party interests, the following situations represent a more structural dimension of this problem.

At a meeting of the Financial Committee, the delegates discussed the annual report of the national “Financial Stability Committee.” This committee comprises members of the Federal Ministry of Finance, BaFin, and the Deutsche Bundesbank. This conglomerate of the statist financial executive submits an annual macroeconomic analysis with respect to the stability of the German financial sector to the federal parliament. For reasons of anonymity, we do not name the exact year under review here, which would allow for an inference to the participants of the meeting, and merely discuss the general structure of the report.

Typically, the report contains an assessment of both the overall risk situation of the financial sector and specific areas. For this year, the report detects no significant systemic risk in the German financial sector but identifies areas with a systemic potential. During the party statements it comes to the following situation:

A delegate comments: “We read this report less accurately from year to year. I can imagine that we are losing sight of the systemic risk.” For him, reasons for this are a prolonged period of low interest rates, a potential neglection of the safety of individual borrowers in macro-prudential valuations, and the danger of a burgeoning real estate bubble. In the second round of statements another delegate replies to this: “We are still reading the report very carefully.” She further articulates that, following the findings of the report, no acute dangers would become apparent.

In another committee meeting, the members discuss the IMF's “Global Financial Stability Report,” which, besides a dedicated focus on global finance, is equivalent to the first report. Here, too, the report identifies risk that is not acutely systemic but has the potential to become so.

A delegate comments the report: “We criticized similar elements [omitted] years ago, but did nothing, with the well-known consequences. The current risk pricing is incorrect.” He proceeds, announcing that compared to parliaments in other countries, there were two variants: “Variant A. Everything is assumed to be safe, while others already criticize. Variant B. We are too critical, while others say that everything is safe.”

At first glance, these examples seem to represent a routinized political exchange of evaluations. One delegate criticizes an inaccurate reading, another contradicts it. A third delegate in another session finds an incorrect “risk pricing” on the basis of another report. However, a closer look at these and other situations reveals that systemic risk in the parliamentary field under study is translated into a political heuristic that, in turn, generates a problem: When is the threshold reached to mitigate systemic risk? The two reports of the (supra-)state financial executive serve as the calculative baseline for the committee. However, in the course of the parliamentary evaluation and collective debate, the dimensional complexity of systemic risk is reduced. It is transformed to make it politically manageable in order to interpret it along diverse political interests. It is the inaccurately read report and not the calculated measure that leads to the interpretation of losing sight of systemic risk. This creates the impression of a dichotomous view in the sense of an existing or non-existing systemic risk.

In principle, there is no reason to act if no acute dangers were identified in the two situations. Nevertheless, the inaccurate reading and “incorrect risk pricing” is criticized, which subsequently indicates a danger: namely, whether the threshold for political action to mitigate the systemic risk has already been reached. This threshold problem is explained by the third delegate, who asks to what extent, compared to other parliaments, one's impression of the global financial sector is fraught with uncertainty. This implies the possibility of various thresholds along various national parliaments. A collective misevaluation of such thresholds of systemic risk could even threaten the more genuine political objects at risk mentioned above.

This dichotomization is, in turn, criticized by representatives of the statist financial executive, which calculate and oversee systemic risk. This is described in the following quote by a macro-prudential analyst. Elsewhere in the interview, she formulates a demarcation line: although the financial executive carries out the analyses, decisions on the use of instruments to mitigate systemic risk are taken by politicians and are thus often normative.

Because this zero or one decision, either we have risks or we do not have risks, I do not think it makes sense. We have a continuum with zero at one end and lots of risks at the other.… We try to quantify, to determine how high are the risks? What would be the damage if this or that happens?… But the world model, which focuses on one definitive threshold … would be presumptuous.

These state practices assess finance as a systemic risk, thus marking one side of the risk-related relationship between finance and statehood. As shown, to construe finance as a risk object also leads to translations (Callon, 1986) between financial-executive and parliamentary state fields that subsequently challenge established relations within the state field. The primarily quantitatively oriented finance-executive (although not exclusively) builds the calculative basis of systemic risk, whereas in parliamentary processes it becomes subject of a qualitative-collective re-calculation (Callon and Law, 2005) and complexity reduction in order to make it politically manageable.

### Political Risk

In contrast to systemic risk, political risk is a much less elaborated, institutionalized, or discursively charged concept. Apart from this, the logic is equivalent when economic actors address an entire state field and construe it as a risk object that threatens financial objects at risk. Thus, the maintenance of liquidity and the generation of profit margins serve as a general category here (Luhmann, 1993). In the case of a bank, this can concern, for example, decisions for or against buying or selling financial assets, consequences for the risk portfolio, or evaluations of the bank's risk management by customers.

Political risk is usually assigned to a range of country risks and can be understood as “a strategic, financial, or personnel loss for a firm because of non-market factors” (Stephens, 2016), like political or public instabilities (Busse and Hefeker, 2007; Jakobsen, 2010). In this environment, business consultancies offer intellectual expertise, country profiles, or quantitative scores on political risk (Howell, 2014; Hulsman, 2018). Research on political risk in view of relations between finance and statehood elaborates the connection between political risk and the issuance of government bonds (Huang et al., 2015). It is also investigated how the interplay of an international and, likewise, national organized financial regulation is perceived as risk for banks in their home country and their foreign subsidies (Kudrna and Gabor, 2013).

#### State as Risk Object

Political risk is anything but a novel category in finance. However, the crisis-driven reflections of the financial actors under study reveal a new quality of this concept. Rather, this is related to changed ascriptions of risk to statehood in the home country or the European level than to foreign nations. Financial field respondents describe current statehood as countering financial complexities by building up regulatory “counter-complexities.” However, it is not the regulative offense that is crucial for the financial actors under study, but the practical operationalization. In this regard, one banker articulates that “politics is currently, of course, extremely preoccupied with itself,” both nationally and at the European level. This would inevitably lead to “dissipating” situations if contradictory positions must be brought into balance. Another banker reflects that the problem is the missing “understanding of each other” (finance and statehood), whereby it is politicians in particular who do not understand bankers.

That financial risk assessments toward statehood are changing is part of the following statement by the head of a derivatives and structuring department:

In my opinion, politics and economics have very rarely had such a high degree of interdependence as it is now…. Political risk is a completely new risk category that financial market participants have to recognize. We were not used to it before. In the past, political risks consisted of: is the CDU [Christian Democratic Union of Germany] or the SPD [Social Democratic Party of Germany] winning right now? That was definitely not a game changer. With all the preferences you can have here, but that is not what you would say now: So does that mean get out of the EU or not?.… Well, this [Brexit] is a completely different dimension … and of course, I must declare this as a risk factor.

It should be added here that it is primarily the more genuine (financial) economic development and valuation that determines trading in the financial sector under investigation. Thus, it is not (yet) a matter to intensively capture every political decision and analyse it in order to buy or sell financial assets. However, financial actors identify a variety of new sources for dangers with respect to statehood, which, corresponding to their self-understanding, must be declared as risk. For this, three particular areas come into question, each addressing different dimensions of statehood as a risk object: central banks as politicized actors, state regulation and supervision of the financial sector, as well as the emergence of a new contingency of political action.

##### Central Banks

I think it [ECB] goes, with respect to its tasks, how it fulfills and lives them, far beyond a central bank. I am not sure if I find this quite chic that it is fulfilling both supervisory and monetary politics. Because there is a recognizable conflict of objectives. The central bank mandate has politicized it.

As in this statement by a banker, recognizing that the ECB is a political actor, thus marking the first dimension. For this, the financial actors under study predominantly refer to the era of low interest rates and bond purchasing programmes. Contrary to the formal independence of the ECB granted by mandate (which is, in turn, emphasized by the politicians interviewed), financial actors assume that the ECB exerts political influence and exceeds its mandate if, for example, it is indirectly involved in the financing of state budgets in the Eurozone. In the long run, this hybrid function of the ECB (Braun, 2020) would, according to the actors, lead to legitimacy crises. For them, the danger is that with ongoing purchase programs and low interest rates, the potential “drop height” will also increase, as a banker describes it. If this comes to an end in the future, the financing conditions of the public and private actors supported by this “broadband antibiotic” (another banker) would be massively worsened. This, in turn, would increase the risk of a financial bubble and downward spiral.

##### Supervision and Regulation

The second dimension concerns the statist financial executive and, thus, supervision and regulation of finance. In principle, financial actors confirm the relevance of stronger regulatory intervention in the aftermath of the subprime crisis, which would have made the system more “robust”. However, new uncertainties also arise. Frequently, the surveyed financial actors refer to the “counter-complexities” passed by the new supervision and regulation regime in reaction to the likewise complex financial operations in banks or insurance companies. The result is a problematic interplay between the “internal” complexity of financial actors as “risk managers” and the regulatory complexity imposed from the “outside.” This is marked in the following statement by a bank's risk manager: Even regulators claim that risk management should remain the competence of the financial actors, while, at the same, due to regulatory offensives, various other actors are involved in these processes. This results in procedural uncertainty (Dequech, 2011) becoming a danger.

The guidelines of the supervisory authority on the subject “deal with your risks, that's your job,” the proportionality approach [double proportionality], MaRisk [Mindestanforderungen an das Risikomanagement/minimum requirements for risk management] and so on. It was meant well but implemented disastrously, because as soon as a bank started to think for itself, the methodologists [from state supervisors] came and said “You can't do what you're doing right now.” Then the associations came along, then politics came along, then technology came along, and so on and so forth.… You have no chance that, through the use of sense and rational thinking, something is made suitable for you and you have a good feeling to label the result as right and effective for controlling.

##### Political-Parliamentary Operations

The third dimension concerns uncertainty with regard to political-parliamentary operations. At times, for the surveyed financial actors, such decisions are neither rationally comprehensible nor transparent, thus these are erratic. Then, political risk is seen in light of “risk events,” which refers to the major political events of the 2010s, like the Brexit referendum. Hereto, a banker describes:

One of the major political risks we are seeing right now is the question, is the UK leaving with or without a treaty? It's extremely difficult to determine that right now. And … everybody is floating around.

As a first reaction, financial actors formulate generic questions to an emerging danger. They do so because the uncertainty of the consequences of such a political contingency is not compatible with their established (quantitative, see below) knowledge and risk routines. For instance, for the head of the derivatives and structuring department, Brexit is viewed as a “field experiment” that challenges the typical “laboratory” of financial calculus (Beunza and Stark, 2004).

I don't know, what happens when a country leaves the EU. What will happen with the rest, or with the country itself, with the currency, or with the economy? Every experiment here is a field experiment. It is not reproducible like in a lab.

### Financial Objects at Risk

The actors and actions that render statehood as risk object also make salient a number of objects at risk that revolve around, in particular, liquidity and margin-seeking.

Concerning the danger of a prolonged period of low interest rates and ECB bond purchasing programmes, interest rates as the epitome of finance activity are generally endangered. However, our interviewees also refer to more direct consequences with respect to their daily risk taking. They speak of a “distortion” of market prices resulting from quantitative easing:

With no doubt, the risk prices on the market are not the right prices. This is quite logical if the [ECB] buys up significant shares of covered bonds and government bonds. Then, I do not wonder if the price no longer reflects the risk. It is as simple as that. And the question is also, if they would stop that, where would the price be?

Thus, it is less problematic that the bond purchases cause prices to fall. What rather follows from this observation, among others, is the problem that financial actors depend on the continuous flow of “true” prices that reflect the market collective: here, the price “no longer reflects the risk.” This applies to both the risk assessment of one's active market positions as well as the various quantitative risk calculations that use such price flows as basis for calculating, for instance, the risk of a current balance sheet due to the simulation of historical and current market volatilities.

Focusing on supervision and regulation as risk objects, financial actors basically face the challenge of complying with or deviating from regulatory requirements (which, in turn, marks the risk object of the regulators under study). Especially in times of low interest rates and declining margins, this can mean to migrate to less regulated markets or deliberately violating (smaller) regulatory requirements (Thiemann and Lepoutre, 2017). However, as shown above, the new plurality of “external” actors intervening in “internal” financial risk management can lead to control problems. Thus, the risk manager, perhaps under the impression of resignation, recommends that his department should be duplicated:

Normally, you should duplicate this department. And do all the work twice. Once the way oversight wants it and once the way you want to manage it.

We conclude that, with respect to the impression of the surveyed financial actors, current political decision-making is, at times, erratic and contingent. What is threatened here is the quantitative fundament of financial risk management, like the use of econometric models and algorithms (MacKenzie and Millo, 2003; Beunza and Stark, 2004; Chiapello and Walter, 2016; Lange et al., 2016; MacKenzie, 2018). Consequently, this further threatens the idea to guide and control investment decision on this basis. First, to consider Brexit as a “field experiment” confronts established calculative practices with a lack of “statistical track record,” as the head of structuring and derivatives further describes. This means that the possibilities of common statistical associations are dwindling with respect to such events, beginning with statistical fundamentals like the correlation between two variables. This is part of the following statement. Equivalent to the calculation of systemic risk, a “qualitatization” (Callon and Law, 2005) of risk practices becomes obvious. This is striking insofar, because this actor is massively surrounded by quantitative calculations but addresses the formation of such a qualitative mode of risk calculation with respect to Brexit:

Here [Brexit], I have to figure out what are the risks? Simply, it is hard to anticipate and, consequently, I cannot tackle this with the usual methods and quantify it to say: “We have just regressed it. That and that comes out of it.” But rather, I have to classify it qualitatively.

These qualitative classifications to develop “imagined futures” (Beckert, 2016) are hardly standardized and operate much more strongly via the formulation of generic questions. Finding answers is conducted by means of narrative-textual descriptions, scenario analyses, and also affective registers, for example through the attribution of market sentiments. Thus, in this division, the statistical-mathematical repertoire for analyzing such political decisions is largely absent. Qualitative (e)valuation practices are in use, which are otherwise atypical in this area.

However, the quantitative calculus is not disappearing. There is still a continuous flow of economic indicators and correlatives. Particularly in areas like quantitative engineering, to deviate from the quantitative standard would contradict their calculation principle. However, as the quantitative engineers underline in our study, to identify concrete risk types and their causes is not crucial, rather it simulates different market volatilities and their effects on price formation. In doubt, the ranges of such volatilities are artificially widened in respective models in order to calculate how a bank's assets would behave in such a case.

Besides these important differences across calculation types, we observe that the quantitative calculus is questioned by the financial actors under study. Not least, this concerns the probability paradigm as fundament of risk calculation. The following statement by another banker underlies this:

“It is not included in the … common risk models. Just try to assign political action of our time to a probability. If you managed to do so, come back.”

Overall, statehood as a risk object threatens financial objects at risk and challenges established risk practices. Distorted prices as a basic risk signal of market collectives due to the current role of central banks, operational challenges due to regulatory offensives, and an endangered quantitative calculus due to political contingency represent a new significance of the state as a source of financial uncertainty.

## Conclusion

In economic sociology and political economy, reflections on the current era of crises have predominantly addressed financialization as a process promoting (crisis-induced) change in the relations between statehood and finance. However, little attention is paid to relationality as a dedicated perspective to uncover further, not exclusively financialized, interactions and entanglements between finance and statehood. Thus, the present contribution argues for a more in-depth understanding of the dynamics of the finance-state nexus that rests on the distinct, field-specific practices of state and financial actors as well as (changes in) their common and intertwined practices. To do so, we focus, theoretically, on risk as a concept that is dominant in both politics and finance to handle situations involving uncertainty and potentially hazardous consequences of a decision situation. Capitalizing on relational theories of risk and the concept of risk entanglement, we aim at doing justice to our goal of uncovering changes in the relationality of the FSN. Empirically, our contribution draws on data from 68 interviews in the German state and finance fields as well as on 1 year of ethnographic observations of these fields.

Our study of risk entanglement in the FSN reveals how crisis-ridden dynamics in the FSN lead to constellations in which finance and state actors construe each other as risks as well as to emerging practices aimed at addressing these risks. On the one hand, systemic risk, which entails finance as the prime risk object, endangers statist aims at the preservation of power as the fundamental object at risk. Whereas, in the financial executive, systemic risk as a prevailing macroprudential principle is primarily determined by quantitative means, it is collectively and qualitatively re-calculated in the parliamentary domain. Thus, translation problems between these two governance areas entail the danger of not recognizing the threshold for when a crucial level of systemic risk has been reached, which would require immediate intervention to contain it. On the other hand, political risk, entailing statehood as the prime risk object, signals hazards to liquidity and profit, the fundamental objects at risk in finance. When looking at current monetary politics, the regulation and supervision of finance, as well as at political decision-making, financial actors see a new quality in statehood as a dangerous and uncertainty-producing entity that challenges finance's established risk practices.

With regard to these findings, we see a number of potential avenues for future research. Generally, this concerns the scope of our study being limited to the German FSN. Explicitly accounting for supra-state levels (such as the EU) and the global scale of the financial sector seems a promising endeavor for more comprehensively investigating relational risk and risk entanglement. For example, current opportunities as well as restrictions to strengthen financial and fiscal consolidation in the European Union can be conceived of as a case of risk entanglement. In the area of sovereign debt management, policies such as debt mutualisation and the issuing of Eurobonds (see van Aarle et al., 2018; Stoupos and Kiohos, 2021), on the one hand, have potential to reduce political and systemic risk through fiscal communitarisation. On the other hand, this can also lead to new risks and an enlargement of risk entanglement given that communitarisation can also result in problems of moral hazard or free riding. In this context, the analytical focus on the Next Generation EU stimulus package in the wake of COVID-19 seems particularly promising, as this is the first time that EU-wide bonds have been issued (see de la Porte and Jensen, 2021).

Furthermore, elaborating the proposed conceptual framework can strengthen future analyses of the FSN. First, the manifold relations between finance and statehood suggest that risk entanglement is not merely a by-product of relational risk and corresponding risk practices, but is in itself a common risk practice, in the sense that, for example, risk entanglement becomes a specific strategy of securitization. In principle, statehood is also involved in the emergence of systemic risk, for instance, when issuing government bonds as risk assets in finance. In contrast, when finance is involved in regulatory frameworks, for example, it can also affect the formation of political risk. Second, focusing on the dynamics of risk entanglement may reveal a certain circularity. For example, the emergence of political risk could trigger financial risk management, which, in turn, can increase systemic risk, thus necessitating statist risk management, which, in turn, could lead to an acute political risk, etc. Third, future research should look at the handling of risks that do not initially emerge in the FSN, such as COVID-19, but which are likewise assessed by finance and state actors. Thus, it seems important to elaborate how such “risk shocks” affect the more institutionalized forms of risk entanglement between finance and statehood.

Specifically looking at the relations between state and finance fields allows for a better understanding of both the practices that are germane to these fields and those that are shared and common across fields. For such an understanding, theoretical as well as methodological progress in relational analysis is essential, to which we hope to have provided some initial steps.

## Data Availability Statement

The datasets presented in this article are not readily available because confidentiality agreements with the actors under study restrict this. Requests to access the datasets should be directed to markus.lange@soziologie.uni-halle.de.

## Ethics Statement

Ethical review and approval was not required for the study on human participants in accordance with the local legislation and institutional requirements. The patients/participants provided their written informed consent to participate in this study.

## Author Contributions

ML conducted the qualitative data analysis. CS developed the main theoretical implementations. All authors equally contributed to further detail data analysis, theoretical approach, the integration of both parts, contributed to the research design and organization of the study, equally contributed to manuscript preparation, revision, read, and approved the submitted version.

## Funding

This research was funded by Deutsche Forschungsgemeinschaft (Project No: 288540449). We acknowledge support by the Open Access Publication Fund of the Freie Universität Berlin.

## Conflict of Interest

The authors declare that the research was conducted in the absence of any commercial or financial relationships that could be construed as a potential conflict of interest.

## Publisher's Note

All claims expressed in this article are solely those of the authors and do not necessarily represent those of their affiliated organizations, or those of the publisher, the editors and the reviewers. Any product that may be evaluated in this article, or claim that may be made by its manufacturer, is not guaranteed or endorsed by the publisher.

## Footnotes

^1^Following field theory as one guiding perspective in our understanding of risk entanglement, one could also speak of a “field-wide” slump in finance activity. However, we rely on “systemic risk” as the term used in the field under investigation.
